# Developing an Animal Welfare Assessment Protocol for Livestock Transported by Sea

**DOI:** 10.3390/ani10040705

**Published:** 2020-04-17

**Authors:** Emma Dunston-Clarke, Renee S. Willis, Patricia A. Fleming, Anne L. Barnes, David W. Miller, Teresa Collins

**Affiliations:** School of Veterinary Medicine, College of Science, Health, Engineering and Education, Murdoch University, Perth 6150, Australia; reneeswillis@gmail.com (R.S.W.); T.Fleming@murdoch.edu.au (P.A.F.); A.Barnes@murdoch.edu.au (A.L.B.); D.Miller@murdoch.edu.au (D.W.M.); T.Collins@murdoch.edu.au (T.C.)

**Keywords:** live export, cattle, sheep, animal health, animal behaviour

## Abstract

**Simple Summary:**

Australia exports large numbers of live cattle and sheep by sea to many destinations. Increasingly high animal welfare standards are being required of all livestock industries, and reports of substantial mortality events on some voyages have raised public concerns regarding animal welfare. Mortality rates alone do not assure stakeholders that livestock experience adequate welfare throughout the voyage. Determining the animal welfare status of large animal consignments is complex and requires many measures that are focused on the environment and resources provided, and also on how the animals respond to their surroundings. A list of measures, appropriate for use on cattle and sheep that enter the livestock export supply chain, was determined by reviewing three international welfare assessment protocols, and consulting the Australian livestock export standards and an animal health handbook used by shipboard veterinarians and stockpersons. After preliminary testing of the measures on a sheep and cattle voyage, we propose a protocol that is potentially practical and applicable for pen assessments for both species at pre-export and destination feedlot facilities and during sea transport. Proposing a protocol is the first step towards developing a system that evaluates livestock welfare throughout the export supply chain, and will contribute to improved industry transparency.

**Abstract:**

Australian livestock industries face increased scrutiny from animal welfare groups and society, and the long-distance transport of livestock by sea has recently gained particular attention. Other than non-compliance with broad regulatory standards and voyage mortality rates, there is minimal information to ascertain the welfare of exported livestock. There is currently no standardised, validated animal welfare assessment protocol for livestock on-farm prior to live export or when undergoing transport. This study describes a novel assessment protocol suitable for use on live feeder and slaughter animals exported by sea from Australia. Health and welfare indicators for use in the livestock export supply chain were identified by reviewing three internationally recognised animal welfare assessment protocols for livestock; Welfare Quality^®^, AWIN and AssureWel, as well as consulting with industry compliance standards and guidelines. This paper proposes a welfare protocol designed to assess sheep and beef cattle exported by sea from Australia, and incorporates environmental-, resource-, management- and animal-based measures. In collaboration with industry, this welfare protocol can be tested on commercial livestock consignments, and be used for ongoing management, for increased transparency and to provide feedback to operators for continuous improvement.

## 1. Introduction

The sea transport of livestock is an integral part of Australia’s agriculture industry, involving the shipping of over 2.6 million live animals per annum, mainly to the Middle East and South-East Asia [1]. The export process includes sourcing, handling, loading and transporting over many days to weeks, and can have a significant impact on the welfare of transported animals [2]. General public concern about farm animal welfare is well documented [3,4], with 95% of the Australian public identifying farm animal welfare as an issue, while 91% indicated that industry reform is needed to address these concerns [5]. Under Australian export of livestock, many of the welfare breaches that have been reported are linked to the climatic conditions experienced on-board ships which result in poor air quality and heat stress [6,7]. Companies that export livestock from Australia are licenced and must comply with the Australian Standards for the Export of Livestock (ASEL) [8] that cover the sourcing animals, the provision of resources (feed, bedding, water, etc.), and management of livestock (stocking density, veterinary supplies, stock handlers, etc.). 

Previously, mortality rates were the primary animal welfare measure recorded under Commonwealth government regulations [9]. These regulations have recently been reviewed [10] and it has been identified that reporting on additional, animal-based measures is required. Several international farm- and abattoir-focused assurance programs have responded by ensuring that audits incorporate an animal welfare focus [11]. The inclusion of animal-based measures within a welfare assessment protocol requires careful selection, however, as measures must be considerate of the species, the environment that animals experience, and be repeatable and standardised to ensure that multiple assessors provide valid and comparable outputs [12]. Assessments must also be easily integrated into the production system to ensure uptake of the monitoring process. Furthermore, a successful protocol not only assesses the welfare of animals at a specific point in time but also evaluates and highlights the potential risks to welfare [13]. Finally, transparency to all stakeholders, including the public, depends on the visibility of production processes and how these processes impact animal welfare [13].

Current welfare assessment protocols highlight the importance and preference for animal-based indicators over resource- or management-based indicators for on-farm assessments [14]. This is due to the recognition of animals as sentient beings that are capable of experiencing both positive and negative emotions [15]. Animal welfare is multidimensional and encompasses many animal-based factors, including health, the absence of stress and pain, the ability to perform innate behaviours, and affective state [16]. Additional to conventional animal behaviour measures, many animal welfare protocols include Qualitative Behavioural Assessments (QBA) [17,18]. Qualitative Behavioural Assessment is a methodology involving a dynamic, holistic observer assessment of the animal as it responds to its environment [15]. Previous studies using groups of observers to describe livestock body language have shown that this technique is useful when assessing the stress and behaviour of livestock during transport [19,20,21] and slaughter [22,23]. As QBA is non-invasive and quick to record [20], adapting this method for use in pen-side assessments could become an important tool for the industry. 

Qualitative Behavioural Assessment can involve the use of a fixed term list or free choice profiling method. A fixed term list is deemed to be most appropriate for a commercial setting due to its feasibility for pen-side assessments and analysis and is practical for repeated measures. While consideration of housing facilities and management strategies are essential, genetic variation, early formative experiences and temperament mean that individuals may respond differently to the same environment. Additionally, differences in management practices, specifically animal handling, can result in varied animal responses. Therefore, focusing only on management- and resource-based measures or inputs is constrictive [24]. Importantly, the absence of negative welfare indicators does not necessarily infer that the welfare of an animal is good [25]. Many welfare assessment protocols have been developed for livestock species, with programs focusing on intensive [14,24,26] and extensive [11,12,27] systems. However, it is important to note that not all measures are applicable nor suitable for use in all production systems [11].

For welfare assessments to be effective and acceptable to all stakeholders, they must incorporate measures that are meaningful with respect to animal welfare, be practicable, and reliable and require minimal resources and personnel time [12,15,28]. As found by the Welfare Quality^®^ project, practicality demands that measures must be quick and easy to record [24] and efficiently integrated into the current regime of stockpersons, shipboard personnel or feedlot workers. Due to the multidimensional nature of animal welfare, there is no single indicator that can be considered to comprehensively evaluate the welfare state of an animal [29], and measures are context specific [11]. The livestock export industry provides a unique challenge for the collection of welfare assessments. First, there are variations in husbandry and handling due to differences in management and resources between pre-export and receival feedlots, as well as on different ships. Second, there are environmental variations as animals are usually transported through a range of climates and seasons (travelling between hemispheres). Third, there are also variations at an animal level with different species, breeds, sexes, ages and weight classes often being exported in one shipment. 

This study aimed to develop a welfare assessment protocol to be used for observing penned animals at different stages of the export supply chain for feeder and slaughter animals. This welfare assessment protocol could then be applied to improve animal welfare reporting, increase industry transparency, promote better welfare by outlining areas that can be improved and contribute to assuring the industry’s social licence to operate.

## 2. Materials and Methods

Three existing international welfare assessment protocols for sheep and beef cattle were reviewed to identify measures applicable to livestock throughout the export supply chain. The AWIN and AssureWel protocols were developed for sheep and beef cattle under both indoor and outdoor housing systems, while Welfare Quality^®^ was developed for intensively housed beef and dairy cattle. The four principles and 12 criteria outlined by the Welfare Quality^®^ project [17] were adopted and the AWIN [18] and AssureWel protocols were reviewed for relevant sheep [30] and cattle [31,32] measures. As our protocol was developed for livestock that were largely pasture-raised and subsequently placed under regulated intensive housing conditions during sea transport, it was important to consider any additional resource-, environmental- and animal-based measures that may be applicable. Therefore, the Australian Standards for the Export of Livestock (ASEL) [8], the “Meat and Livestock Australia (MLA) Veterinary handbook for cattle, sheep and goats” [33] and a survey about the importance of welfare indicators (completed by members of the public and livestock export industry workers) [34] were consulted. Two of the reviewed protocols applied QBA, with 20 and 21 terms used by the Welfare Quality^®^ and AWIN protocols, respectively. The definition of QBA terms used were modified from these protocols in combination with additional studies [29,35,36].

Review of the above resources provided a long list of 64 measures for pre-testing. Each putative measure was considered by the research team, applying knowledge and experience of the live export industry, in terms of feasibility for the export context (time efficient), validity (relevance to the species when animals are in supply chain) and reliability (ability to produce consistent results when performed at different timepoints or by different assessors). Many of the indicators had only been tested in production systems and we, therefore, considered whether the measure was also valuable for the feedlot or ship setting. Where there was no suitable animal indictor for a criterion provided by the resources, we then considered other resource- and environmental-based measures. This refinement process resulted in 74 measures to be tested (Table 1). 

Data collection sheets for both sheep and beef cattle were developed using the smartphone application Kizeo forms [37] to allow standardised pen-side data collection. Pre-testing of the protocol was conducted for one cattle consignment in the southern hemisphere 2017 winter, and for one sheep consignment in Autumn 2018. At all locations (pre-export facility, voyage and destination feedlot), measures were recorded by one observer, viewing the animals in their pens. The number of animals in pens ranged between 7 (vessel)–100 (pre-export facility) for cattle and 30 (vessel)–200 (pre-export facility) sheep. For a measure to be considered as applicable to sea transport, it was required to be valid to the focal species, practicable to be collected pen-side and require only easily portable equipment. At the end of preliminary testing, the measures that were used and deemed applicable to the sea transport of feeder and slaughter sheep and cattle, but also considerate of the assessment conditions, including the environment and husbandry practices of the industry or the type of livestock exported, were included in our proposed protocol. 

## 3. Results and Discussion

Although welfare assessments are routinely performed by proficient stockpersons [38], standardising and documenting these evaluations is uncommon. Reporting on welfare assessments using standardised methods will help identify welfare risks and build industry transparency and enable greater stakeholder understanding of the on-board livestock experience. 

### 3.1. Practicability of Assessment under Commercial Conditions

The practicality and adoption of a welfare protocol is dependent on the ability for the measures to be collected quickly, easily and repeatably [24]. As the protocol will be designed for use by live export industry workers, it is important that all necessary measures are captured using non-invasive methods and in a timely manner. The time taken to complete the assessment for each pen of livestock differed depending on the location. At each location, numerous pens were observed, with the pre-export facility and destination feedlot assessments taking approximately 10–15 min per pen, including static management- and resource-based factors (stocking density, number of feed and water troughs) and livestock factors (weight, class, fleece length). Subsequent on-board pen assessments took less than the minimum of 10 min for Welfare Quality^®^ [39], the 25 min required for AssureWel protocols [30,31,32] and 40 min for AWIN [18].

Measures were categorised under the four welfare principles: good feeding, good health, good housing and appropriate behaviour. 

### 3.2. Good Feeding

For housed cattle, the management of feed supply and access to feed and water are critical [11]. Feed and water measures are also considered important on export vessels because such resources need to be produced (water) or carefully managed (feed) throughout a voyage. Measures related to feeding were placed under the categories “absence of prolonged hunger/appropriate nutrition” and “absence of prolonged thirst”. Body Condition Score (BCS) is considered an important measure for both species and is regarded as a robust, accepted and preferred measure for evaluating medium to long-term good feeding [11,12,40]. A shorter-term measure of feeding was required for our protocol; consequently, the number of troughs, the amount of feed available and cleanliness of feed troughs were included. The reviewed protocols do not include an animal-based measure to quantify feed intake; therefore, we developed a measure described as Feed Behaviour Score, because feeding behaviour is particularly important as it is informative about the immediate level of hunger, social competition for feed [41], and appetitive response to climatic challenges [42,43,44]. The Welfare Quality^®^ protocol contains additional measures for water provision, including water availability and cleanliness. While ASEL requires export vessels to carry some reserve feed supplies in case of unforeseen extensions to the voyage [8], daily monitoring of these resources is important [33]. Stocking density and pen design may prevent all animals in a pen from accessing feed or water troughs simultaneously. Measures can be monitored to indicate good feed and water access, such as trough fill or contamination (with faeces, saliva or pellet fines). The use and outcome of these resources can be measured by assessing animal behaviour at feeding and gut fill. 

### 3.3. Good Housing

Measures related to housing were placed under the categories “comfort around resting”, “thermal comfort”, “ease of movement”, and “environmental measures”. Many of the variables under these criteria are interrelated and the latter category has been included in the proposed protocol because the environmental conditions on-board require ongoing management and greatly influence animal welfare.

Comfort around resting involves measuring the manure pad depth and moisture level, measures which are captured under our protocol but are missing from the reviewed protocols. Dairy cattle have been observed to prefer to lie on dry surfaces [45], while mud and faecal contamination have also been found to result in poor animal welfare for beef cattle in feedlots, resulting in pen surface management being a priority [46]. On-board livestock vessels, moisture from manure and urine or water supplies can impact the integrity of the manure pad. Therefore, to assess aspects related to thermal comfort of animals during a voyage, the manure pad and ventilation must be monitored. These measures become particularly important during a heat stress event, which leads to increased drinking and, therefore, more urine output, subsequently impacting the consistency of the manure pad and leading to increased local humidity. All protocols include a measure of coat or fleece cleanliness, and the extent of manure coverage of the hind, lower legs and flank. These measures are an important [11] for housed animals, indicating the cleanliness of the floor and bedding [12]. These measures are especially important during heat stress events, as a faecal coat can impede an animal’s ability to thermoregulate and lead to ill-health [46].

The health and welfare of livestock exposed to thermal challenges of varying severity and duration are not well defined [2]. Measuring the internal body temperatures of individual animals is not practical in a commercial setting because entering a pen to collect such measurements can disturb animals; this is inappropriate when the animals are already under thermal challenge. The reviewed welfare protocols assessed thermal comfort by measuring panting scores. Panting is an important heat loss mechanism for cattle and sheep, indicating the animal’s response to increased environmental temperatures, with sustained panting indicating a continued need for the animal to shed heat [2]. Therefore, panting scores provide a non-invasive means to assess the animal’s thermal environment and response. The ability to evaluate panting score level and duration, in combination with health and behavioural outcomes as well as varying environmental conditions, will better inform stakeholders about the impost of thermal stress on animal welfare, and the risk of compromise under differing conditions. 

Our protocol contains many environmental measures, which can vary between ship locations, voyage day and time of the day. Wet bulb temperature (T_WB_) is commonly used as a shipboard measure to incorporate ambient temperature and humidity, both of which are important in determining the heat balance of an animal [46]. During periods and journeys which might expose the animals to hot conditions, the live export industry uses the Heat Stress Risk Assessment (HSRA) Model [47] to determine the stocking density on voyages. The model considers climatic conditions (e.g., predicted and actual in destination ports and en route), animal factors (e.g., class, weight, BCS and fleece or coat length) and ship factors (e.g., ventilation flow and design) [47]. The fleece of sheep and coat length of cattle are recorded as; shorn sheep have lower core and rumen temperature, as well as respiratory rate than unshorn sheep [2,48], while cattle with winter coats are more prone to coat contamination and less able to dissipate heat [33]. In addition to panting score and environmental conditions, other measures of importance during a thermal challenge include ventilation, manure pad, coat/fleece length and contamination.

Ease of movement considers animal body posture and can inform the effect of stocking density and whether a preferred body posture can be achieved. Other than considering factors that relate to live weight and stocking density, the reviewed protocols lack measures that consider this aspect in detail. As social species, cattle and sheep in extensive systems synchronise their feeding and resting behaviour [49,50], with synchrony being proposed as a welfare measure in other livestock systems [51]. Sheep reduce time spent lying when the resting areas are uncomfortable and space allowance insufficient [27]. Observing cattle in different lying positions, such as sternal vs. lateral recumbency, could indicate thermal comfort or discomfort [25] and provision of suitable deck space allowance. Therefore, a reduction in time spent and synchrony of lying may be an indicator of reduced welfare. 

### 3.4. Good Health

Criteria included under Welfare Quality^®^’s health measures are “absence of injuries” and “absence of disease”. Health measures in our protocol were applicable to intensively housed livestock, and included additional health indicators based on conditions detailed in the “Veterinary handbook for cattle, sheep and goats” [33] and industry experience. The measures selected were those recognisable when livestock are observed from outside of the pen, because assessments are designed to be non-invasive. The absence of injuries, such as lameness, skin lesions and wounds can reflect the suitability of the pen environment, sea conditions and how the livestock were handled during land transport and loading or discharge [52,53,54]. Animals that are loaded or discharged quickly have a higher risk of injuring themselves on hard, sharp or slippery surfaces [55]. Some of the measures under absence of disease might also reflect the animal’s environment. For example, ocular discharge and coughing could indicate elevated ammonia levels [56], while nasal discharge could indicate dusty feed causing irritation [33]. Therefore, these animal-based measures provide critical information on the impact of the environmental. “Absence of painful procedures” which normally is used to record specific practices, such as castration and de-horning, was removed from the protocol because these are not performed during the export process.

### 3.5. Appropriate Behaviour

Behavioural measures were placed under categories “expression of social behaviour”, “expression of other behaviours”, “good human–animal relationship” and “positive emotional state”. Change in animal behaviour is often the first and most obvious indicator of a change in an animal’s ability to cope with procedures and the surrounding environment [57]. With the focus of welfare protocols shifting towards the inclusion of animal-based measures, incorporating animal behaviour is a logical step. Unlike the animals assessed under the reviewed protocols, animals in the livestock export chain are exposed to a variety of environments within short time periods; changes in behaviour can, therefore, be indications of the animal’s ability to cope. It is important that behavioural measures can also capture negative, neutral and positive. Behaviour considered to indicate negative well-being include agitation, aggression and pushing [27]. It is also important to consider how restrictive environments not only change natural behavioural patterns, but also the incidence of aggressive behaviour, as these impact on surrounding animals and interactions with humans. In dairy cattle, changes in lying behaviour and a positive correlation between the incidence of agonistic behaviour and large avoidance distances between humans and cattle have been observed in a confined environment [14].

Our protocol includes measures that capture positive well-being, including play, exploration and social- and self-grooming; however, these may occur infrequently, reducing their use within a welfare protocol [15]. Play behaviour is considered to be an indicator of a positive emotional state [27], only evident when the animal’s needs are met. While play can, therefore, indicate good welfare, the incidence of play is naturally more common in younger animals [25]. Animals within the export supply chain may have little opportunity to display positive behaviour or be mature and less likely to engage in play behaviours, these measures could provide useful information about positive well-being that is not captured elsewhere. Methods that capture positive emotional would be beneficial, enabling positive welfare states to be identified outside of the presence of these infrequent behavioural events. 

In addition to quantitative measures of activity, qualitative measures of behaviour were also included. Two of the reviewed protocols incorporate QBA, using a fixed list of approximately 20 terms for farm assessments. For our protocol, a list of 10 terms for sheep and 12 for cattle were chosen to capture the demeanour of penned livestock during sea transport, while being time efficient to collect (Table 2). These selected terms were selected as they were deemed to capture the full range of demeanour observed during preliminary testing and included four negative (anxious, dull, frustrated and uncomfortable) and four positive terms (content, happy, inquisitive, settled), along with two terms that can be described as neutral and maybe context specific (active, alert). Applying QBA to an export context is novel but the method can be reliably measured at a group level. By focusing on animal expressions, observers become more sensitive to how the animals are coping and communicating within their environment, which can be invaluable when determining welfare states [15].

For our protocol, it was decided that a more in-depth evaluation of the human–animal relationship was required. This is due to Australian livestock generally originating from extensive grazing systems before entering the live export supply chain, whereupon they are confined in close quarters and are frequently exposed to human interaction. The quality of the human–animal interaction is well known to impact animal welfare and productivity [58]. To evaluate the human–animal relationship, the flight distance between the observer and animals in the pen was measured as approach was made. In our protocol for cattle, the reactions of the animals to the researcher’s presence (e.g., turning of head to view the researcher vs. displaying aversion to their presence) was also recorded.

### 3.6. Excluded Measures

Measures that were relevant only to extensive livestock or dairy (i.e., access to pasture and health of lactating cows), were excluded (11 sheep and 13 cattle measures) (Table 3) as they were not applicable to the livestock export context due to the differences in environment, animal (e.g., lactating cow) and timing (on-farm procedures). Although dairy cattle are also transported by sea, they are not lactating nor in late gestation, thus the development of our welfare protocol focused on slaughter and feeder animals. 

### 3.7. Additional Measures

Our protocol incorporates novel measures that have not been applied by existing protocols, in order to capture environmental (e.g., sea swell, pen area, ventilation and air quality) and animal-based (e.g., breed, class, polled vs. horned) factors that vary within and between voyages (Table 4). Specifically, sea swell is a welfare-relevant measure particular to the voyage environment. It is not known whether livestock suffer motion sickness at sea, but it was identified as a welfare concern by industry and the public. Recent land-based studies on a small sample of sheep detected an aversion to periods of simulated sea motion [59,60]. Hence, recording animal-based outputs during different sea conditions can better inform stakeholders about the effect of sea swell on animal welfare.

### 3.8. What is the Application of this Protocol?

To address societal concerns surrounding the continuation of the export of Australian livestock, the monitoring of animal welfare and transparency throughout the supply chain is needed [7,9,61]. Before this is achievable, the development of a protocol that incorporates scientifically valid methods of assessing animal welfare is required. As reviewed above, there have been many welfare protocols developed for indoor and outdoor farmed livestock; however, the live export process presents challenges not experienced by these land-based production systems. Therefore, we present a protocol that encompasses the important environmental-, resource- and animal-based measures applicable to the environments and feeder and slaughter sheep and cattle during sea transport. Novel animal-based measures previously not incorporated in existing protocols have been proposed and may be applicable to other farming systems and species. Sharing our protocol, it may assist other scientists and industries in the development of further protocols. 

## 4. Conclusions

Identifying measures that may be applicable to the welfare assessments of sheep and cattle transported from Australia by sea is the first step in developing a protocol for welfare monitoring. Uniquely, the livestock export process provides numerous environmental-, resource-, management- and animal-based challenges, and thus, requires additional criteria not used by other protocols. We identified and included measures that were practical for the livestock export context and omitted those that were not relevant. Whether all of the listed measures should be routinely used requires validation over several voyages of data capture. For a representative welfare assessment of large stock numbers, it is likely that numerous measures must be taken at multiple time points along the journey. It is also likely that some measures will be more important than others under certain conditions, such as during periods of high heat. The protocol must be flexible to accommodate use in the varied phases of the livestock export supply chain and climates. The exact parameters for the application of the protocol will be established once it has undergone piloting in the industry. It is predicted that the proposed welfare protocol will provide the foundation for standardised data collection that can be analysed for industry self-improvement and for building greater stakeholder understanding. 

## Figures and Tables

**Table 1:** Welfare measures from existing protocols for cattle and sheep, and for the proposed Australian livestock export protocol. Colour indicates measures that are species specific; blue—cattle only measures, green—sheep only measures, and black—applicable to both species.

Welfare Principle.	Welfare Criteria	Welfare Measure	Welfare Quality^®^	AWIN	AssureWel	Proposed Live Export Protocol
Good Feeding	Absence of prolonged hunger	• Body condition score	☑	☑	☑	☑
		• Fodder ration availability				☑
		• Feeding regimen				☑
		• Roughage availability				☑
		• Amount of food left in troughs				☑
		• Feed contamination				☑
		• Trough access				☑
		• Feed behaviour score				☑
		• Animals eating				☑
	Absence of prolonged thirst	• Water provision/availability	☑	☑		☑
		• Water troughs				☑
		• Cleanliness of water points	☑			☑
		• Water flow	☑			
		• Function of water points	☑			☑
		• Animals using watering points	☑			☑
Good Housing	Comfort around resting	• Animals standing and lying				☑
		• Time needed until animals to lie down	☑			
		• Animals colliding with housing equipment during lying down	☑			
		• Animals lying partly or completely outside of lying area	☑			
		• Cleanliness of flank/upper legs and lower legs	☑	☑	☑	☑
		• Manure pad depth				☑
		• Manure pad moisture				☑
	Thermal comfort	• Panting	☑	☑		☑
		• Air quality	☑			☑
		• Access to shade/shelter		☑		☑
		• Fleece length				☑
		• Coat length				☑
		• Wet bulb temperature (°C)				☑
		• Dry bulb temperature (°C)				☑
		• Relative humidity (%)				☑
		• Heat stress condition				☑
	Ease of movement	• Stocking density	☑	☑		☑
		• Pen area				☑
		• Animals in pen				☑
		• Horn length				☑
		• Live weight	☑			☑
		• Breed and class				☑
		• Draft				☑
	Additional environmental conditions	• Location				☑
		• Sea swell (category)				☑
		• Air quality (score 1–5)				☑
		• Air movement/ventilation performance (score)				☑
		• Noise (score)				☑
Good Health	Absence of injuries	• Lameness	☑	☑	☑	☑
		• Integument alterations	☑			
		• Lesions		☑	☑	☑
		• Wounds				☑
		• Swellings			☑	
		• Hair loss			☑	☑
		• Broken tails			☑	
		• Leg injuries		☑		
		• Mobility			☑	☑
	Absence of disease	• Coughing	☑			☑
		• Sneezing				☑
		• Vocalisations				☑
		• Belching				☑
		• Scabby mouth				☑
		• Faecal egg count			☑	
		• Ocular discharge		☑		☑
		• Ocular lesions				☑
		• Pink eye				☑
		• Nasal discharge	☑			☑
		• Ocular discharge	☑			☑
		• Respiratory quality	☑	☑	☑	☑
		• Pneumonia treatments			☑	☑
		• Diarrhoea/scours	☑			☑
		• Bloat	☑			☑
		• Hollow sides				☑
		• Illthrifty				☑
		• Downer/unable to stand	☑			☑
		• Skin irritation/itching			☑	
		• Animal needing further care			☑	☑
		• Offspring born				☑
		• Aborted pregnancies				☑
		• Animals moved to hospital pen and reason				☑
		• Animals euthanised				☑
		• Mortality	☑	☑	☑	☑
Appropriate behaviour	Expression of social behaviour	• Agonistic behaviour	☑			☑
		• Cohesive/social behaviours	☑			☑
		• Social withdrawal		☑		
	Expression of other behaviours	• Stereotypy		☑		
		• Excessive itching		☑		
		• Posture				☑
		• Activity				☑
	Good human–animal relationship	• Avoidance distance	☑		☑	☑
		• Familiar human approach test		☑		
		• Animal behavioural response to human				☑
	Positive emotional state	• Qualitative Behavioural Assessment	☑	☑		☑

**Table 2:** Qualitative behavioural assessment terms used by existing protocols and proposed for the livestock export protocol. Colour indicates measures that are species specific, with blue indicating cattle only, green indicating sheep only, and black indicating measures that are applicable to both species.

Term	Welfare Quality^®^	AWIN	Measures Included for Livestock Export and Definition	
Active	☑	☑	☑	Energetic, lively, characterized by busy or lively activity (body movement and actions)
Aggressive		☑		
Agitated	☑	☑	☑	Restless, fidgety, worried or upset
Alert		☑	☑	Animals are fully aware, attentive, vigilant (how engaged the animal is with the surrounding environment)
Anxious			☑	Animals are worried, nervous, uneasy, increased vigilance
Apathetic	☑	☑		
Assertive		☑		
Bored	☑			
Bright		☑		
Calm	☑	☑		
Content	☑	☑	☑	Animals are appeased, comfortable, at ease, satisfied with its environment and needs are met
Defensive		☑		
Distressed	☑			
Dull			☑	Animals are inactive, indifferent to their environment, lacking interest
Fearful	☑	☑		
Friendly	☑			
Frustrated	☑	☑	☑	Animals are annoyed, impatient, prevented from achieving something
Happy	☑		☑	Animals are positively occupied, showing enjoyment
Indifferent	☑			
Inquisitive	☑		☑	Animals are positively interested, curious, showing active investigation
Irritable	☑			
Listless		☑	☑	Animals are lacking energy, uneasy and not engaging with surrounding environment
Lively	☑			
Playful	☑			
Positively occupied	☑			
Relaxed	☑	☑		
Settled			☑	Animals are quiet, relaxed, calm, not tense
Sociable	☑	☑		
Subdued		☑		
Tense		☑		
Vigorous		☑		
Wary		☑		
Uneasy	☑			
Uncomfortable			☑	Animals are troubled, showing signs of physical discomfort, unease irritation

**Table 3:** Welfare measures from existing protocols for cattle and sheep that were not included within proposed export protocol. Colour indicates measures that are species specific; blue—cattle only measures, green—sheep only measures, and black—applicable to both species.

Welfare Principle	Welfare Criteria	Measure	Reason for Exclusion
Good Feeding	Appropriate nutrition	Lamb mortality ^b^	Our protocol is not designed to focus on breeding animals.
Good Housing	Ease of movement	Access to outdoor loafing areas/pasture ^a^	Livestock do not routinely have access to loafing areas or pasture during the export supply chain.
		Presence of tethering ^a^	Beef cattle are not tethered during the export supply chain
	Comfort around resting	Cleanliness of udders ^a^	Our protocol is not designed to focus on breeding animals. Coat cleanliness and stocking rate are included.
Good Health	Absence of injury	Hoof overgrowth ^b^	Measure included under “lameness”.
	Absence of disease	Faecal soiling ^b^	Measure included under “coat cleanliness”.
		Fleece loss ^c^	Measure included under “lesions”.
		Fleece quality ^b,c^	Fleece length is included, poor fleece quality to the detriment of welfare would be included under the measure “lesion”.
		Mucosa colour ^b^	This requires handling of individual sheep which is not performed.
		Udder lesions ^b^	Our protocol is not designed to focus on breeding animals. Udder lesions causing poor welfare would be included under “lesion”.
		Hair loss ^a^	Measure included under “lesions”.
		• Caesarean and assisted calving ^a,c^ • Dystocia ^a^ • Calf/heifer survival ^c^	Our framework is not designed to focus on breeding animals. Measures would be included under calf/lamb born or aborted pregnancy.
		Cull and casualty cows ^a^	Animals deemed “not fit to load” are not exported by sea.
		Mastitis ^b,c^	Our protocol is not designed to focus on breeding animals.
	Absence of pain induced by management procedures	• Eat notching ^b,c^ • Disbudding/dehorning ^a,c^ • Tail docking ^a,b,c^ • Castration ^a,b,c^	These procedures occur on farm before sourcing for export.
Appropriate behaviour	Expression of other behaviours	Access to pasture ^a^	Cattle do not routinely have access to pasture during the export supply chain.

^a^ Welfare Quality^®^, ^b^ AWIN, ^c^ AssureWel.

**Table 4:** Measures with descriptions for the proposed protocol for the Australian livestock export. Measures, unless specified, are to be recorded at a pen level.

Good Feeding	Good Housing	Good Health	Appropriate Behaviour
- Body condition score (score 1–5) - Fodder ration availability (%body weight/head/day) - Feeding regime (increased roughage/reduced pellets, restricted fodder, maintenance, above maintenance, ad lib) - Roughage availability (grams/head/day) - Amount of food left in troughs (empty, some crumbs left, ¼ full, ½ full, full) - Feed contamination (clean, some fines, majority fines, some faeces/saliva/mould, marked faeces/saliva/mould) - Trough access (m) - Feed behaviour score (not observed, disinterested, some interest, keen, jostling, aggressive/smothering) - Water availability (hours/day) - Water points (number of troughs/bowls) - Cleanliness of water troughs (not observed, clean, mild dust/fodder/saliva, moderate faeces/dust/fodder/saliva, marked contamination + % of water points contaminated) - Function of water points (notes recorded on broken, leaking, etc.)	- Coat/fleece cleanliness (all are clean and dry, some with belly, flanks and legs covered, most with belly, flanks and legs covered, muddy/dung contaminated/damp, heavily soiled/wet, filthy/very wet) - Manure pad depth (cm, average of pen) - Manure pad moisture (description of majority of pen = dry and dusty, firm, tacky, high moisture, sloppy, flooded (caused by sea spray, water leak, rain)) - Panting score (% of pen per score with cattle = 0–4.5, sheep = 0–5) - Air quality (clear air, slight smell, moderate smell, marked smell, strongly irritant) - Air movement (still air, slight breeze, moderate breeze, windy, strong wind) - Noise (quiet, medium, noisy, very noisy) - Access to shade/shelter (none, trees/wind break, shade cloth/partial shade, roof, roof and walls) - Fleece length (off shears, short fleece <25 mm, med fleece 25–35 mm, heavy fleece >35 mm) - -Coat length (short, short-medium, medium, medium-heavy, heavy) - Wet bulb temperature (°C) - Dry bulb temperature (°C) - Relative humidity (%) - Sea swell (no swell, low swell (<2 m), moderate swell (2–4m), 4 heavy swell (> 4m), phenomenal/confused swell) - Stocking density (m^2^/head) - Pen area (m^2^) - Animals in pen (count) - Horn length (cm, polled and short, polled and long) - Live weight (kg) - Breed and class (breed and sex) - Draft (evenly drafted, 1–2 animals above pen average, 1–2 animals pen below pen average, some variation of size, marked variation) - Location (farm, pre-export facility, ship, destination feedlot, lairage)	- Lameness (no. animals) - Lesions (no. animals) - Wounds (no. animals) - Hair loss (no. animals) - Unable to stand (no. animals) - Coughing (nil, less than 1 per min, >1 per min individual animal, >1 per min multiple animals) - Sneezing (nil, less than 1 per min, >1 per min individual animal, >1 per min multiple animals) - Vocalisations (nil, less than 1 per min, >1 per min individual animal, >1 per min multiple animals) - Belching (nil, less than 1 per min, >1 per min individual animal, >1 per min multiple animals) - Scabby mouth (no. animals) - Ocular lesions (no. animals) - Pink eye (no. animals) - Nasal discharge (% of pen) - Ocular discharge (% of pen) - Diarrhoea/scours (no. animals) - Bloat (no. animals) - Hollow sides (% of pen) - Illthrifty (no. animals) - Animal needing further care (no. animals) - Offspring born (no. animals) - Aborted pregnancies (no. animals) - Animals moved to hospital pen (no. animals and reason) - Animals euthanised (no. animals and reason) - Mortality (no. animals and reason)	- Posture (standing, lying, % of pen) - Agonistic social behaviour (% of pen) - Affiliative social behaviour (% of pen) - Activity, % of pen: - chewing fence - self-grooming - allo-grooming - eating - drinking - ruminating - visual - exploring environment - play - abnormal behaviour - Flight distance (m distance at which human observer enter flight zone when approaching pen) - Animal behavioural response to human (HAR), % of pen: - animals that stand - animals that look - animals that retreat - animals that approach - Qualitative behavioural assessment (score of 0–100 per pen, per term)
